# Tunable dual-AAV sparse labeling of PV^+^ retinal ganglion cells enables single-neuron projection by fMOST

**DOI:** 10.3389/fncir.2025.1740624

**Published:** 2026-01-12

**Authors:** Lingbo Zhou, Gao Tan, Yu Li, Man Yuan, Sen Jin, Wenhui Zhang, Qitian Wang, Yin Shen

**Affiliations:** 1 Eye Center, Renmin Hospital of Wuhan University, Wuhan University, Wuhan, Hubei, China; 2 Zhongmou Therapeutics Co., Ltd., Wuhan, Hubei, China; 3 Frontier Science Center for Immunology and Metabolism, Wuhan University, Wuhan, Hubei, China

**Keywords:** AAV, fMOST, PV^+^ RGC, single cell reconstruction, sparse labeling

## Abstract

**Introduction:**

Sparse and bright labeling of retinal ganglion cell (RGC) is essential for correlating single-cell morphology with brain-wide visual circuitry. This study aimed to develop a cell-type-specific, sparse labeling strategy for parvalbumin-expressing RGCs (PV^+^ RGCs) in the transgenic mouse retina using recombinant adeno-associated virus (rAAV) and to map the whole-brain projection patterns of single PV^+^ RGCs via fluorescence micro-optical sectioning tomography (fMOST).

**Methods:**

A cell-type-specific dual AAV system was employed, co-packaging a Cre-dependent Flpo plasmid and an Flpo-dependent enhanced yellow fluorescent protein (EYFP) plasmid. Key parameters-including the mixing ratio of core plasmids (ranging from 1/100 to 1/1000), gene copy number of Flpo and EYFP (single versus double), and AAV serotype (AAV2.2 versus engineered AAV2.NN)-were systematically optimized. Transduction efficiency and labeling sparsity under each condition were compared. Whole-retina-to-brain imaging was performed using fMOST on samples injected with the optimal condition (AAV2.2-double-1/1000), enabling the reconstruction of complete axonal trajectories of individual PV^+^ RGCs from the retina to the brain.

**Results:**

The sparsity and signal intensity of labeled RGCs varied significantly with the core plasmid ratio, AAV serotype, and gene copy number. The engineered AAV2.NN serotype increased transduction efficiency and labeling density under equivalent conditions, which facilitated the morphological subclassification of PV^+^ RGCs into ON, ON-OFF, and OFF types based on their stratification relative to ChAT bands. Axonal projections of single PV^+^ RGCs were successfully traced to the superior colliculus (SC), dorsal and ventral lateral geniculate nuclei (dLGN/vLGN).

**Discussion:**

This viral labeling platform effectively resolves the classical trade-off between sparsity and signal intensity, providing a robust methodology for whole-brain mapping of individual RGC projections. The approach establishes a practical foundation for future mechanistic and therapeutic studies investigating subtype-selective vulnerability in RGCs.

## Introduction

The retina is a part of the central nervous system (CNS), and its intricate neural circuit structure underlies visual information processing (Corso-Díaz et al., 2018). Understanding the morphological characteristics of various types of neurons in the retina, such as the dendritic branching, axon projections, and synaptic connections they form, is critical for uncovering the fundamental principles of visual perception (Wernet et al., 2014; Ibrahim et al., 2019; Helmstaedter et al., 2013). Such insights are also essential for the diagnosis and treatment of retina-related diseases, such as glaucoma and diabetic retinopathy (Mead and Tomarev, 2016; Wang et al., 2020; Goyal et al., 2023).

To overcome the limitations of bulk labeling, sparse labeling techniques have been developed (Ku et al., 2016). These methods utilize genetic or viral tools to stochastically label only a small subset of neurons within a tissue, significantly reducing the complexity of neuronal imaging (Jefferis and Livet, 2012; Jamal et al., 2020; Veldman et al., 2020). Sparse labeling has thus become a powerful strategy for studying neuronal morphology and connectomics, allowing researchers to obtain clear and precise data on individual neuronal structures (Li et al., 2010; Kuramoto, 2019; Han et al., 2018). However, current sparse labeling methods, including recombinant adeno-associated virus (rAAV) delivery and transgenic mice models, face significant challenges (Qiu et al., 2022). A primary technical hurdle is the balance between labeling density and signal intensity (Lu, 2021; Matsumoto et al., 2024). Diluting viral vectors to achieve sparsity often results in faint labeling, compromising the visibility and traceability of labeled neurons. Conversely, increasing viral titers to enhance brightness typically results in excessive labeling density, obscuring individual neuron details. This trade-off complicates efforts to achieve both optimal sparsity and sufficient signal strength. Despite the advantages of AAV vectors, such as low immunogenicity and sustained transgene expression (Wu et al., 2025), efficiently achieving sparse labeling of parvalbumin-positive (PV^+^) RGCs remains a significant challenge.

To address these challenges, we developed an optimized AAV-based sparse labeling strategy integrated with fluorescence micro-optical sectioning tomography (fMOST). This innovative approach enabled the effective sparse labeling of individual PV^+^ RGCs and facilitated comprehensive mapping of their whole-brain projection patterns. Additionally, by screening a range of AAV serotypes, we were able to classify distinct subtypes of PV^+^ RGCs based on their labeling profiles. This research offers a novel methodology for analyzing retinal neural circuits at a higher resolution. Furthermore, the insights gained and experimental tools developed in this study provide a foundation for advancing gene therapies targeting retinal degenerative diseases, paving the way for new therapeutic strategies.

## Materials and methods

### Construction of virus vector

HEK293T cells were seeded at a density of 7.0 × 10^6^ cells per disk 24 h prior to transfection. The transfection involved employing a combination of an adenoviral helper plasmid, a Rep/Cap plasmid, and a plasmid mixture comprising DIO-Flpo and FDIO-EYFP or DIO-Flpo-Flpo and FDIO-EYFP-EYFP in specific ratios, delivered using PEI Pro (Cwbio, CW9309M). After a 72-h incubation period, the cells were harvested and collected by centrifugation. Viral particles were liberated through the application of a high-salt lysis buffer, followed by five cycles of freezing and thawing. To eliminate genomic DNA and residual plasmids, Benzonase (Sigma, 9025-65-4) was added prior to the precipitation of proteins, including AAVs, with polyethylene glycol (PEG, Aladdin, P103734) for 1 h. The precipitated PEG pellet was resuspended overnight, and AAVs were subsequently purified using an iodixanol gradient to eliminate contaminating proteins and empty capsids. The virus-laden iodixanol fraction was concentrated and underwent buffer exchanged through Amicon Ultra-15 filtration tubes (Merck, UFC910024). The concentrated AAV solution was sterile, filtrated, aliquoted, and stored at −80 °C for later use. The viral titer is determined using real-time quantitative PCR (qPCR) and adjusted to 5.0 × 10^12^ vg/mL.

The nomenclature for dual AAV vectors adheres to the format: AAV2.X (where X represents the serotype) + Gene copy (single/double indicating the gene copy number) + ratio (the mixing ratio of Cre-dependent Flpo plasmid to Flpo-dependent enhanced yellow fluorescent protein). For instance, AAV2.2-single-1/100 indicates that this dual AAV vector was produced by co-packaging DIO-Flpo and FDIO-EYFP at a ratio of 1/100 with the AAV2.2 serotype.

### Animal model and intravitreal injection

Experiments were conducted using 6–8 weeks old PV-Cre mice (weighing ∼20 g). All animal procedures were performed in accordance with Animal Ethics Committee of Wuhan University (MRI2024-LAC010). Mice were anesthetized (1.25% avertin, 20 μL/g i.p.), and then intravitreal injection was performed using a microsyringe (Hamilton, #65). The injection volume was 1.5 μL per eye, and the injection site was located 0.5 mm posterior to the limbus, mouse was injected into the right eye only for fMOST. After the injection, mice were kept in a warm environment until fully awake.

### Retinal flat-mount and immunofluorescence staining

Five weeks after injection, mice were deeply anesthetized and then underwent cardiac perfusion with ice-cold 0.1 M phosphate-buffered saline (PBS), followed by fixation with 4% paraformaldehyde (PFA). Ocular globes were carefully extracted and further fixed in 4% paraformaldehyde for 45 min before dissecting the intact retina for retinal flat-mount preparation. The optic nerve was transferred into a sucrose solution for gradient dehydration (at 4 °C). Subsequently, frozen sections were prepared at a thickness of 30 μm. Both retinal flat mounts and sections were washed with PBS and blocked overnight in a solution containing 4% BSAT at 4 °C. Immunofluorescence staining was performed using anti-GFP antibody (Abcam, ab13970) and anti-ChAT antibody (Millipore, AB144P), with all steps conducted at 4 °C. Images were acquired using laser scanning confocal microscopes (Zeiss LSM 880 and Leica Stellaris 5 WLL) and a high-resolution rapid fluorescence microscope (Leica Thunder).

### Tissue preparation for fMOST

The mice were deeply anesthetized and subjected to cardiac perfusion using ice-cold 0.1 M PBS followed by 4% PFA. Post-extraction from the cranial cavity, the brain and eyes were fixed in 4% PFA at 4 °C for 24 h, followed by a 24-h rinse in PBS. The brain and eyes were then prepared for resin embedding.

The samples underwent a graded dehydration process through a sequential series of ethanol solutions at concentrations of 50%, 75%, 95%, and 100%, with each step lasting 2 h. Following dehydration, the specimens were infiltrated with LR-White resin in increasing concentrations (50%, 70%, 85%, and 100%), each for 2 h. They were then immersed overnight in 100% LR-White resin overnight for 36 h at 4 °C, with the resin solution refreshed after 12 h. The final polymerization of the brains was conducted in a vacuum oven set at 38 °C for 24 h. The 100% LR-White resin was prepared by combining 100 g of LR-White with 0.24 g of ABVN as a polymerization initiator.

### fMOST imaging and data processing

The LR-White resin-embedded brains were imaged using the fMOST system (Wuhan OE-Bio Co., Ltd.). Each brain was mounted with the fMOST automated data acquisition system, which comprises a 473 nm laser, a 40× water immersion objective (Olympus, N2667700), and a time delay and integration charge-coupled device (TDI-CCD) for signal detection. Semi-thin coronal sections of 2 μm thickness were sequentially imaged from anterior to posterior, with the data acquisition phase extending over 4–5 days per sample. This meticulous process generated approximately 6,500 coronal sections, facilitating the creation of a comprehensive brain dataset. The raw images, with a resolution of 0.35 μm × 0.35 μm × 2 μm, were processed using specialized software for stripe stitching and brightness normalization. The processed images were then used for 3D reconstruction using Imaris software. The resulting brain dataset was downsampled and aligned to the Allen Reference Brain Atlas, using the Common Coordinate Framework version 3 (CCFv3) for standardized spatial orientation.

### Data analysis and statistical processing

The data were processed using ImageJ and Aivia software. The images and fluorescence intensities were processed or analyzed by ImageJ. To ensure more reasonable normalization of fluorescence signal intensity, we first standardized the imaging parameters for capturing fluorescent images to avoid signal intensity deviations caused by differences in exposure time, gain, etc. All images were obtained under the same microscope settings. Then we standardized the method for fluorescence signal quantification:

1. Open ImageJ software and load the prepared image files into the software.
2. Click on Image→Adjust→Brightness/Contrast and set the Max parameter to 255.
3. Click on Analyze→Tools→ROI Manager.
4. Use selection tool to choose the EYFP-positive somata.
5. Click the Add button in the ROI Manager to add the selected regions.
6. Once all regions have been added, click the Measure button to perform calculations. The results, including parameters such as Area and Mean gray value for each region, will be automatically generated for subsequent statistical analysis.
7. To ensure data accuracy, perform the same experiment three times or more and record the corresponding values, the specific *n*-values are indicated in the legends of the corresponding images.

Finally, apply the normalization formula: subtract the average gray value of the background (background mean gray value) from the average gray value of the target region to complete the fluorescence intensity correction.

The number of fluorescent cells in retinal flat mounts refers to the total count of all EYFP-positive cells in each whole retina, for each group, we analyzed the retinas from five different mice. For each retina, we selected multiple EYFP-positive RGC somata as regions of interest and calculated the mean fluorescence intensity per retina; these per-retina values were then used for statistical analysis. Statistical analysis was performed using GraphPad Prism 9.0. One-way analysis of variance (ANOVA) was used for comparisons between groups, followed by Tukey’s *post-hoc* test for multiple comparisons. Data are presented as mean ± standard deviation (SD), and differences with *p* < 0.05 were considered statistically significant (ns = not significant, *p < 0.05, **p < 0.01, ***p < 0.001, ****p < 0.0001).

## Results

### Screening of dual plasmid mixing ratios for AAV sparse labeling

**FIGURE 1:**
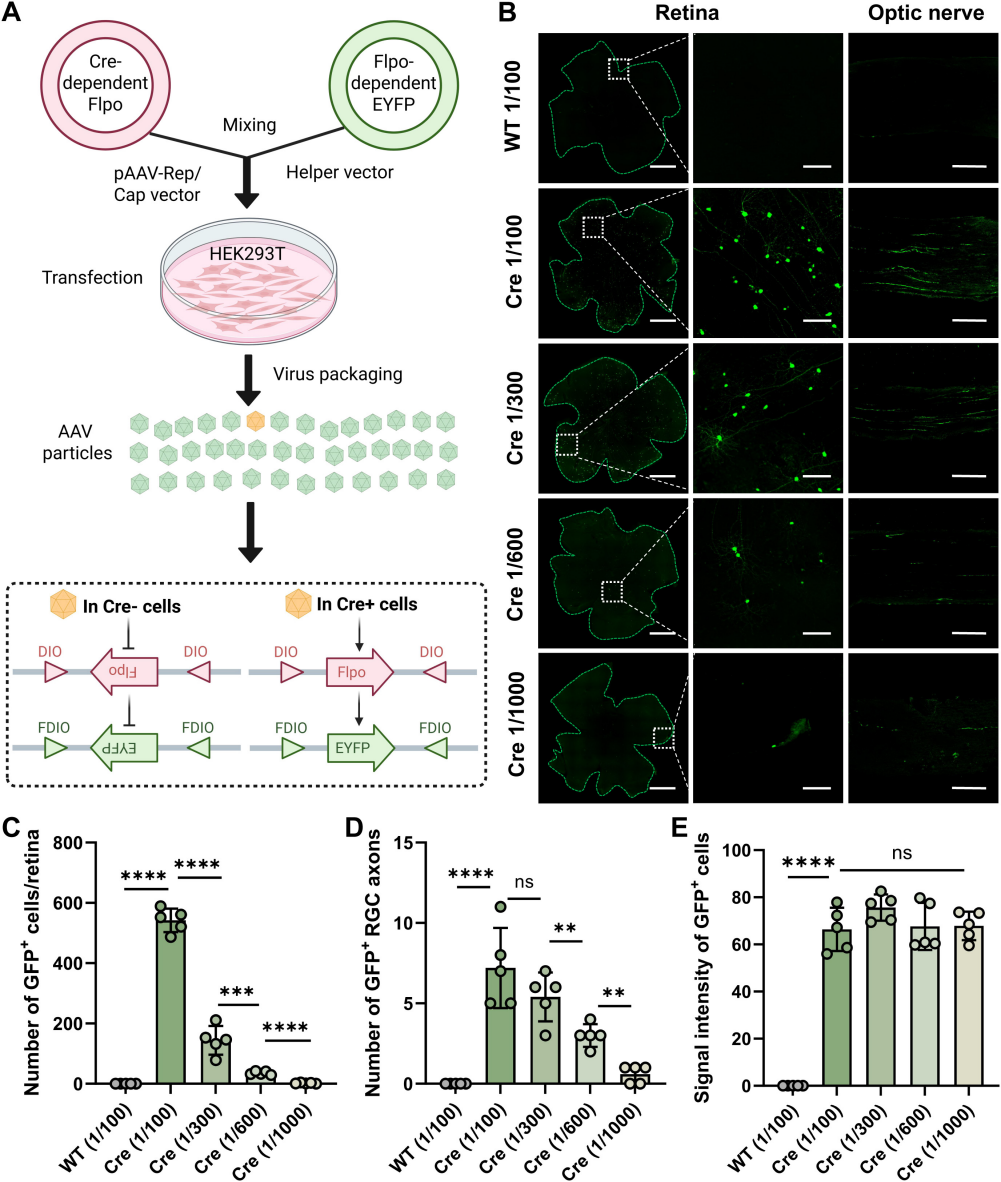
Sparse labeling of RGCs using a dual-plasmid strategy and the resulting labeling patterns at different plasmid mixing ratios. **(A)** Schematic diagram of virus packaging and intravitreal injection in mice. **(B)** The representative image of virus expression in the retina, enlarged view (zoom-in) of dashed square part of left image of retina and optic nerve, 1/100, 1/300, 1/600, 1/1000 represent mixing ratio of the core plasmid. From the left to right, scale bar = 200, 50, 100 μm. **(C)** Statistical chart of GFP positive cell numbers in different groups, *n* = 5 retinas in each group. **(D)** Statistical chart of GFP positive RGC axons in different groups, *n* = 5 optic nerves in each group. **(E)** Statistical chart of signal intensity of cell bodies in different groups, *n* = 5 retinas in each group. Data are represented as mean ± SD, with statistical significance indicated by asterisks (ns = not significant, **p < 0.01, ***p < 0.001, ****p < 0.0001).

We employed PV-Cre mice and a dual AAV system for specifically and sparsely targeting of PV^+^ RGCs. The dual AAV system is consisted of two vectors: a Cre-dependent Flpo plasmid and Flpo-dependent enhanced yellow fluorescent protein (EYFP) plasmid. We obtained four dual AAV vectors products (AAV2.2-single-1/100, AAV2.2-single-1/300, AAV2.2-single-1/600, AAV2.2-single-1/1000) by mixing the Cre-dependent Flpo plasmid and Flpo-dependent EYFP plasmid at four different ratios (1/100, 1/300, 1/600 and 1/1000) during the AAV production. We intravitreally injected these four products into the retina of Cre mice and collected the retina and optic nerve 5 weeks postinjection (Figure 1A). In the WT control mice, EYFP expression in neither retinal cell bodies nor the optic nerve was observed. Meanwhile, the results showed that as the mixing ratio increases from 1/100 to 1/1000, the number of labeled PV^+^ cells progressively decreased (Figure 1B). The number of fluorescent cells in retinal flat mounts is the total count of all EYFP-positive cells in each whole retina. Specifically, the product of 1/100 ratio labeled approximately 542.0 ± 39.2 cells and the product of 1/300 labeled approximately 144.0 ± 47.6 cells, but it is still insufficient to observe the complete morphology of individual cells. The product of 1/600 ratios labeled 35.4 ± 5.8 cells, while the product of 1/1000 ratio resulted in up to 4 labeled cells. Surprisingly, in one retina of 1:1000 ratio group, only a single PV^+^ RGC was labeled (Figure 1C). Accordingly, the number of EYFP-positive optic nerves showed a similar trend to that of EYFP-positive somata, ∼7–8 axons were labeled in 1/100 group, while only 1 axon was labeled in 1/1000 group (Figure 1D). There was no significant difference in the fluorescence intensity of individual retina soma in PV-Cre mice across the different ratio groups (Figure 1E). Thus, we established a novel strategy for sparse and high-brightness labeling of specific types of retinal cells by combining transgenic mice with a dual-plasmid AAV viral system.

### Optimization of sparse labeling conditions by increasing the gene copy number

**FIGURE 2:**
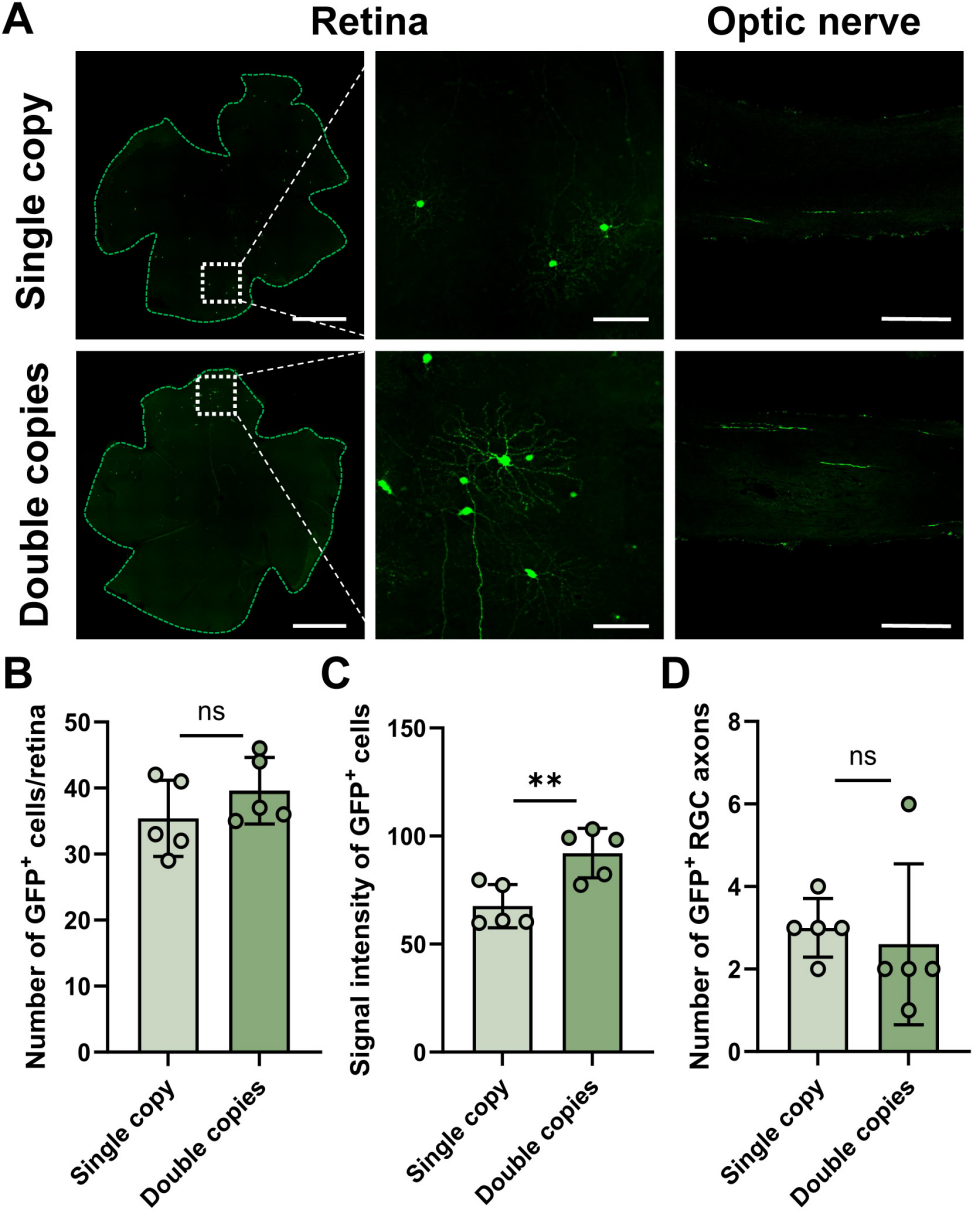
Labeling efficiency of AAV2.2 vectors harboring different transgene copy numbers. **(A)** The representative image of virus expression in the mouse retina, partial enlarged view and optic nerve, single copy and double copies indicate the copy number parameters of the target gene of the core plasmid. From the left to right, scale bar = 200, 50, 100 μm. **(B)** Statistical chart of GFP positive cell numbers in different groups, *n* = 5 retinas in each group. **(C)** Signal intensity of single cell in different groups, *n* = 5 retinas in each group. **(D)** Statistical chart of GFP positive RGC axons in different groups, *n* = 5 optic nerves in each group. Data are represented as mean ± SD, with statistical significance indicated by asterisks (ns = not significant, **p < 0.01).

Next, we systematically optimized the copy number parameters of the target gene in the core plasmid rAAV-EF1a-DIO-Flpo-Flpo-WPRE-hGH-pA and rAAV-nEF1a-FDIO-EYFP-EYFP-WPRE-hGH-pA, within the payload capacity of AAV2.2. By increasing the copy number of Flpo and EYFP within these plasmids, we successfully elevated the expression levels of the EYFP protein, as reflected in the enhanced fluorescence intensity seen in labeled cells (Figure 2A). Interestingly, at a plasmid ratio of 1/600, there was no significant difference observed in the number of EYFP-positive cells or the labeled axons when comparing double-copy with single-copy groups (Figures 2B, D). Nonetheless, the average fluorescence intensity per cell exhibited a significant difference between these groups. Specifically, the mean signal intensity in the single-copy group was 70.2 ± 10.9, whereas the double-copy groups’ intensity was significantly higher 94.1 ± 11.4 (*p* < 0.01) (Figure 2C). This optimization paves the way for more detailed studies on the morphology of RGCs and their projections to downstream brain regions, enhancing our understanding of neural pathways.

### Mapping of individual RGC projections to brain based on fMOST imaging

**FIGURE 3:**
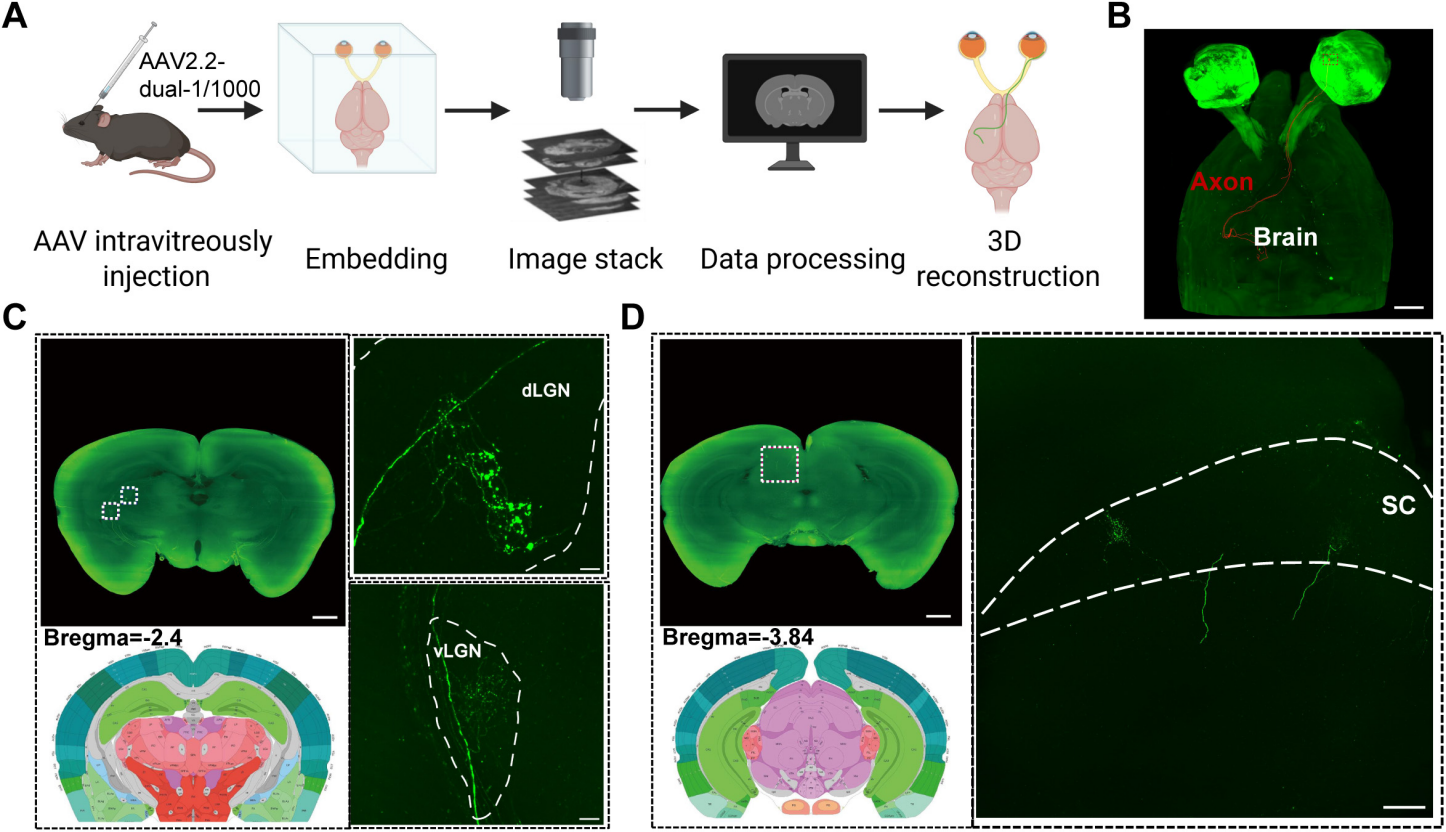
Visualization of RGC projections to brain targets using fMOST imaging. **(A)** Schematic diagram of intravitreal injection in the mouse (right eye), tissue processing and fMOST imaging. **(B)** fMOST imaging of projection from single retinal neurons to brain regions, scale bar = 1000 μm. **(C)** Labeling and enlarged view of projecting nerve fibers in the dLGN and vLGN brain region, bregma = –2.4 mm, scale bar = 1000 μm (left) and 400 μm (right). **(D)** Labeling and enlarged view of projecting nerve fibers in the SC brain region, bregma = –3.84 mm, scale bar = 1000 μm (left) and 400 μm (right).

To elucidate the precise projection patterns of RGC to visual centers at single-cell resolution, we employed fMOST to acquire high-resolution three-dimensional data from the whole brains of transgenic mice subjected to anterograde labeling. Through image registration, neuron tracing, and three-dimensional reconstruction, we successfully reconstructed the complete and morphologically intact axon of a single RGC, along with its distinct projection pathways and terminal branches in all target areas, including the superior colliculus and lateral geniculate nucleus. We performed whole-brain imaging and data reconstruction of sparsely labeled PV^+^ RGC 5 weeks after virus injection to the right eye of PV-Cre mice (Figure 3A). The complete projection patterns of individual neurons were reconstructed (Figure 3B). Computational neuroanatomical analysis revealed that the neuron demonstrated origin locations and distinct projection patterns.

We found that PV^+^ RGCs primarily project to regions such as the superficial layers of the dorsal lateral geniculate nucleus (dLGN), the ventral lateral geniculate nucleus (vLGN) (Figure 3C) and the superior colliculus (SC) (Figure 3D). Supplementary Video 1 demonstrates the complete projection continuously traced from single-cell labeling in the retina to brain regions. We observed the overall trajectory of individual RGC axon upon exiting the optic chiasm. As the axon approach the midbrain and forebrain regions, it exhibits distinct target-specific branching behaviors, directing their projections to SC and LGN, respectively. Notably, we did not observe any RGC axon branches projecting to non-visual nuclei, consistent with their projection specificity. Within the LGN, axon terminals form highly intricate, densely branched arborizations (Figure 3C). Compared to the terminals in the SC, those in the LGN generally display greater complexity, with more branching points, forming a smaller but more compact structural volume (Figure 3D). In summary, our fMOST imaging data reveal a distinct pattern: individual RGC transmit information to both the SC and LGN in a parallel manner via target-specific axonal branching. However, the presynaptic structures formed in these primary visual centers differ significantly in spatial scale, morphological complexity, and subnuclear localization, likely reflecting their distinct functional requirements in processing motion perception (SC) versus pattern and detail vision (LGN-cortical pathway).

### Optimization of AAV serotypes and PV^+^ RGC classification

**FIGURE 4:**
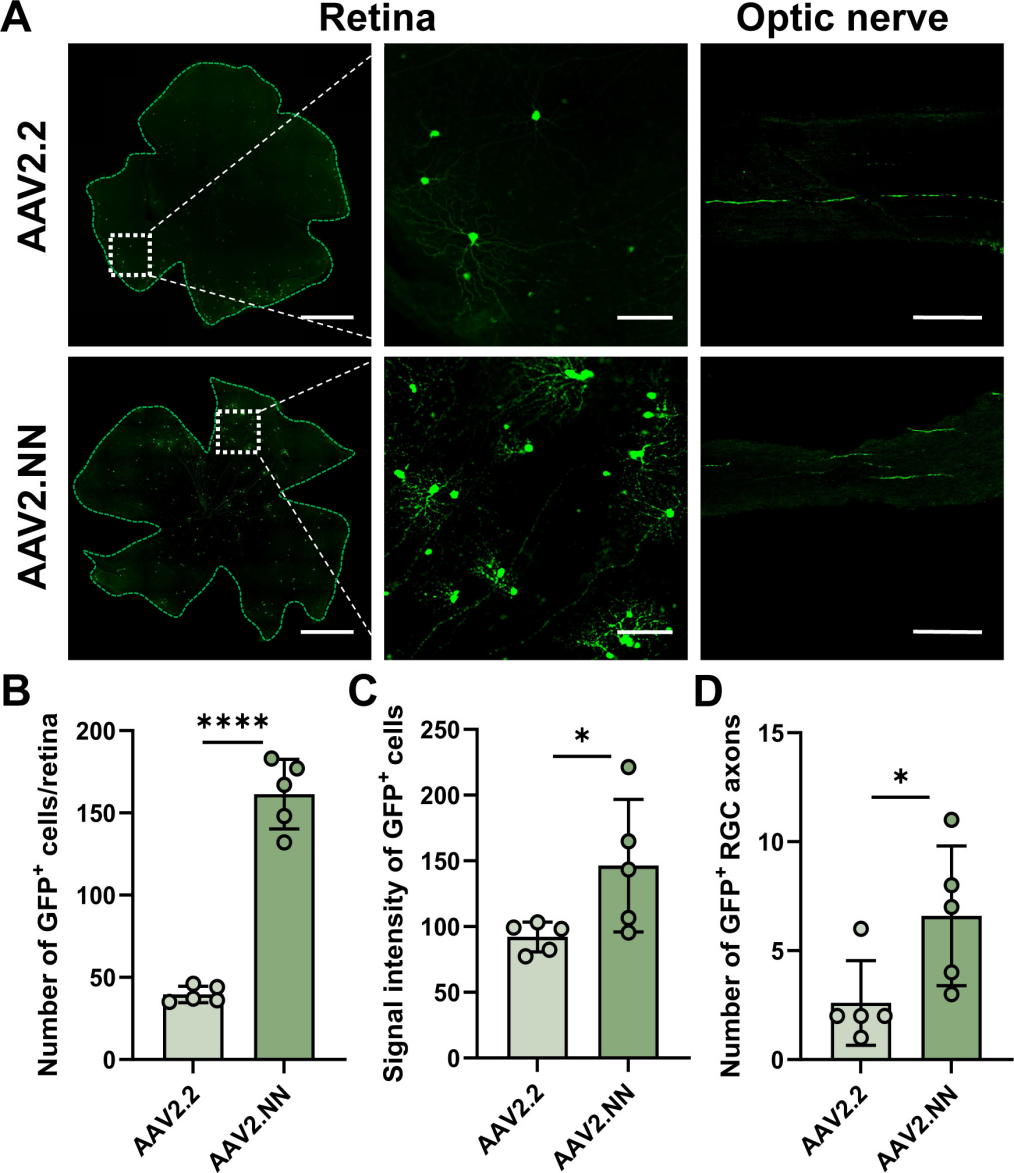
Comparison of labeling efficiency across different AAV serotypes. **(A)** The representative image of virus expression in the mouse retina, partial enlarged view and optic nerve. From the left to right, scale bar = 200, 50, 100 μm. **(B)** Statistical chart of GFP positive cell numbers in different groups, *n* = 5 retinas in each group. **(C)** Signal intensity of single cell in different groups, *n* = 5 retinas in each group. **(D)** Statistical chart of GFP positive RGC axons in different groups, *n* = 5 in optic nerves each group. Data are represented as mean ± SD, with statistical significance indicated by asterisks (*p < 0.05, ****p < 0.0001).

AAV2.2 vectors have been sufficient to meet the needs of studies involving sparse labeling and central projection tracing (Chen et al., 2010). To better delineate neuronal morphology, we selected AAV2.NN as a candidate, which exhibits stronger penetration of the inner limiting membrane in the retina and a larger payload capacity compared to AAV2.2 (Pavlou et al., 2021). We found that the RGC projections labeled by AAV2.NN were more distinct and denser under the same condition compared to that of AAV2.2 (Figure 4A). Whether the number or the fluorescence intensity of labeled cells, AAV2.NN is greater than AAV2.2 (Figures 4B, C). The number of axons also exhibits a corresponding trend (Figure 4D). Therefore, we performed morphological classification of PV^+^ RGCs labeled with AAV2.NN.

The classification of RGC subtypes is instrumental in understanding parallel processing of visual information at the retinal level. In optic nerve degenerative diseases like glaucoma, different RGC subtypes demonstrate varying susceptibility to damage (Denniss et al., 2025; Liu et al., 2023). Recognizing this selective vulnerability is essential for developing targeted neuroprotective therapies. Furthermore, ensuring that regenerating axons in optic nerve regeneration correctly reach their target brain regions relies heavily on their subtype identity.

Recent authoritative studies have classified mouse RGCs into more than 40–50 transcriptomic subtypes (Goetz et al., 2022; Sanes and Masland, 2015), highlighting the complexity and diversity of these cells. In the retina, there is a specific stratified correspondence between the dendrites of ON and OFF RGCs and the dendrites of starburst amacrine cells (SACs) within the inner plexiform layer (IPL) (Sun et al., 2015; Chen et al., 2021; Guadagni et al., 2016). The dendrites of ON RGCs are primarily distributed in the inner sublayer (sublamina b) of the IPL, located at approximately 40% depth (measured from the ganglion cell layer, GCL), overlapping with the dendritic band of ON-type SACs (Supplementary Video 2; Baden et al., 2016; Sanes and Masland, 2015). Conversely, the dendrites of OFF RGCs are concentrated in the outer sublayer (sublamina a) of the IPL, at a depth of about 77% (measured from the GCL), corresponding to the dendritic band of OFF-type SACs (Supplementary Video 3). The dendrites of ON-OFF cells extend into both the ON and OFF sublayers (Supplementary Video 4; Baden et al., 2016).

**FIGURE 5:**
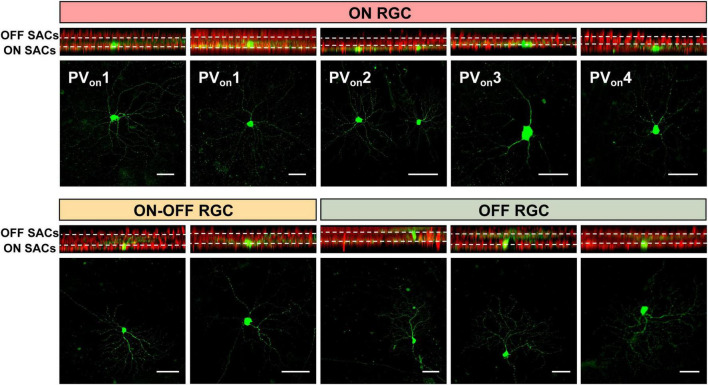
Classification of PV^+^ RGC subtypes based on sparse labeling and morphological analysis. Z-stack layered scanning of the retina with x-z overlay and x-y overlay images. The white dashed lines represent the ChAT (choline acetyltransferase, marker of SACs) labeling schematic, with the upper part indicating ON and the lower part indicating OFF, PV_on_1–4 represents four different types of ON PV^+^ RGCs, scale bar = 20 μm.

As shown in Figure 5, PV^+^ RGCs can be subdivided based on their dendritic positioning within SAC synapses into three types: ON, ON-OFF, and OFF (Yi et al., 2012). Morphologically, ON cells can be further classified into distinct subtypes: PV_on_1, characterized by a large soma and a large dendritic field (large parasol-like), probably with fast conduction speed, primarily responsible for motion detection and luminance information (Goetz et al., 2022; Sanes and Masland, 2015); PV_on_2, featuring a small soma and a small dendritic field (small parasol-like), which might have slower conduction speeds and is mainly involved in high spatial resolution and fine vision (Goetz et al., 2022); PV_on_3, having a large soma with a small dendritic field (sunflower-like); and PV_on_4, distinguished by dendrites exhibiting bilateral symmetry (butterfly-like). This detailed classification and understanding of RGC subtypes provide a foundation for exploring their functional roles and developing interventions that address specific vulnerabilities and regeneration strategies.

## Discussion

We present a practical framework for celltype-specific sparse labeling in the adult retina that reconciles sparsity with brightness and enables brain-wide single-neuron reconstruction. By independently tuning plasmid ratio and gene copy number, we obtain single PV^+^ RGC labels suitable for fMOST without losing visibility. The observed target-specific collateralization to SC/LGN and distinct terminal morphologies highlight pathway specialized presynaptic architectures at single-neuron resolution. Relative to prior sparse-labeling approaches (Lin et al., 2018; Ibrahim et al., 2019), our dual-AAV design requires only a single Cre driver line and standard intravitreal delivery, facilitating adoption. AAV2.NN further broadens use cases by boosting efficiency for subtype surveys and mesoscale mapping.

Sparse labeling is essential for resolving detailed neuronal morphology at the single-cell level (Costa et al., 2016). By optimizing the ratio of the viral core plasmids, we maintained the labeling density within an ideal range, effectively minimizing signal overlap that often arises from excessive labeling. This methodological framework offers a valuable reference for investigating other sparsely distributed neuronal populations. Furthermore, the implementation of a double-copy strategy–by increasing the copy number of core viral components–enhanced labeling clarity, thereby improving the accuracy of neuronal projection tracing. Such advances are critical for elucidating the structural and functional architecture of neural circuits. The application of fMOST technology further permitted the analysis of brain-wide connectivity with single-cell resolution. Our observation of topologically organized projections aligns with findings reported by Jiao et al. (2025) in hypothalamic neurons, suggesting that such organizational principles may be a general feature of brain network architecture. The selection of an appropriate AAV serotype is critical for achieving high transduction efficiency in the retina. Our results indicate that the engineered serotype AAV2.NN transduces RGCs with significantly greater efficiency than the conventional AAV2.2, corroborating recent reports (Zhang et al., 2025). This enhancement is likely attributable to specific mutations in the viral capsid proteins, which improve affinity for cell surface receptors and facilitate viral internalization and intracellular trafficking (Weinmann et al., 2022; Zhang et al., 2025). These improvements in AAV-mediated gene delivery not only support precise neuronal labeling in neurobiology research but also hold therapeutic potential for vision disorders involving RGCs (Zhang et al., 2022; Tribble et al., 2014; Nimkar et al., 2025). Continued refinement of capsid engineering may further extend the utility of this approach to other neuronal subtypes and tissues.

Nonetheless, our study has several limitations. First, all experiments were performed in adult mice, which precluded the examination of development dynamics in PV^+^ RGCs projection patterns (Solomon et al., 2018; Beck et al., 2005). The whole eye–brain level single-neuron tracking system is used to provide a representative example of the long-range projection pattern of a sparsely labeled PV^+^ RGC. We processed the fMOST data by overlaying 25 consecutive 2-μm sections to generate a refined 3D reconstruction. This processing improved the visualization of RGC axonal arborizations and yielded clearer videos showing coronal (Supplementary Video 5) and sagittal (Supplementary Video 6) views of the soma and dendrites in the retina, and the axonal projections in the SC, vLGN and dLGN (Supplementary Videos 7–9). This reconstruction illustrates the feasibility and resolution of our approach, but it does not imply that all PV^+^ ON, OFF, and ON-OFF subtypes share the same projection pattern (Attallah et al., 2021).

Future research directions may include: (1) developing more specific promoters to enable selective labeling of RGC subtypes, a systematic comparison among ON, OFF, and ON-OFF subtypes will require additional, subtype-specific reconstructions; (2) integrating optogenetics with *in vivo* calcium imaging to investigate functional properties of distinct projection subtypes; and (3) establishing disease models to assess alterations in PV^+^ RGC projections under pathological conditions. From a technical standpoint, optimizing algorithms for fMOST data processing will be a key future focus. Isotropic resolution recovery techniques based on deep learning, such as Self-Net, have already demonstrated significant potential (Bastug et al., 2024; Fotaki et al., 2022; Menze et al., 2024). Finally, our findings open new avenues for treating retinal degenerative diseases. For example, AAV vectors could be engineered to deliver neurotrophic factors specifically to PV^+^ RGCs, promoting neuronal survival and axonal regeneration (Rodger et al., 2012; Cen et al., 2017; Yungher et al., 2017). Sparse labeling and neuronal reconstruction–encompassing the precise three-dimensional mapping of somata, axons, and dendrites–remain foundational techniques for realizing these goals (Abdellah et al., 2018).

## Data Availability

The original contributions presented in this study are included in this article/Supplementary material, further inquiries can be directed to the corresponding author.
